# From the Outgoing Editor

**DOI:** 10.3390/jintelligence9040049

**Published:** 2021-10-09

**Authors:** Paul De Boeck

**Affiliations:** 1 Department of Psychology, Ohio State University, Columbus, OH 43210, USA; deboeck.2@osu.edu; 2 Faculty of Psychology and Educational Science, KU Leuven, Tiensestraat 102, B-3000 Leuven, Belgium

The *Journal of Intelligence* was founded in 2013, eight years ago. Our aim was to offer a broad and flexible open-access journal that moves forward the study of human intelligence: the basis and development of intelligence, its nature in terms of structure and processes, and its correlates and consequences, also including the measurement and modeling of intelligence.

We have now published 252 articles on a broad variety of topics related to the domain of intelligence and cognition: cognitive plasticity, academic achievement, social cognition, personal intelligence, the SAT, general intelligence, Geary’s mitochondrial functioning theory of general intelligence, and many more. Looking back at 2013, it was both a jump into limbo and a leap of faith. I am especially thankful to Earl Hunt and Susanne Jaeggi, and to Wendy Johnson for their invited articles in the very first issue, and to Joe Rodgers for the very first special issue, on the Flynn effect. They largely reduced the feeling of being in limbo and provided the journal with a head start. I am thankful to the associate editors and the members of the editorial board. They were a justification of the faith. The associate editors Andrea Hildebrandt, Gizem Hülür, Andrew Conway and Matthias Ziegler are the mainstay for the journal, and many members of the editorial board have sent us their thoughts and have promoted the journal. The first editorial was entitled “Intelligence, Where to look? Where to go?” The authors of the many articles have each provided us with relevant ideas and findings to answer the questions “where to go?” and “where to look”, and so many collaborative reviewers have evaluated the submissions, with helpful suggestions for the authors of the submissions. 

Research on intelligence and related abilities and skills has many aspects. The journal has published a broad range of articles, in the methodological and substantive sense. We have also been open to less common types of articles: conceptual contributions, promising first steps to develop tests for new concepts, discussion and commentary articles. Quantitative approaches have always been important in the study of intelligence. I believe there are two rather new alternatives for the established factor model approaches: network models and cognitive models (e.g., the drift diffusion model), and our journal has contributed to their dissemination. On the other hand, the established factor models and SEM approaches and response time approaches have also led to prominent contributions. Among the 10 most cited articles, there are three on the bifactor model, two on network models, two on response times, and one on the diffusion model. We also have published many articles on related abilities and skills, such as creativity (one article in the top ten cited), social intelligence, emotional intelligence, and more. Although not easy, we have also tried to bridge the gap with education.

As the outgoing editor, stepping down this month, I realize the work is not complete. I am grateful for the opportunity I was given to be editor. I have had fun, except when submitted manuscripts had to be rejected, and the faith in “leap of faith” has also played an important role over the years. Intelligence is an intriguing but risky topic because intelligence is a complex concept with many facets, a concept without a consensus definition, experts have largely different opinions, and there are some difficult controversies in the study of intelligence.

I am glad that Andrew Conway is willing to take over as editor-in-chief. He has ample editorial experience and research expertise in the field of intelligence and cognition. He was associate editor of our journal, of *Journal of Experimental Psychology: General* and *Journal of Cognitive Psychology.* His research is based on an integrated approach combining cognitive science, psychometrics, and neuroscience, with a mix of empirical and methodological work (measurement of working memory capacity, attention) and theoretical work (process overlap theory). I wish him and the associate editors all the best in their further development of the journal. I believe they are in good hands with the publisher and the journal staff, and I am sure the journal is in good shape with the new editor and his editorial team.

